# Corset: enabling differential gene expression analysis for *de novo* assembled transcriptomes

**DOI:** 10.1186/s13059-014-0410-6

**Published:** 2014-07-26

**Authors:** Nadia M Davidson, Alicia Oshlack

**Affiliations:** Murdoch Childrens Research Institute, Royal Children’s Hospital, Flemington Road, Parkville 3052, Melbourne, VIC Australia; Department of Genetics, University of Melbourne, Melbourne, VIC Australia

## Abstract

Next generation sequencing has made it possible to perform differential gene expression studies in non-model organisms. For these studies, the need for a reference genome is circumvented by performing *de novo* assembly on the RNA-seq data. However, transcriptome assembly produces a multitude of contigs, which must be clustered into genes prior to differential gene expression detection. Here we present Corset, a method that hierarchically clusters contigs using shared reads and expression, then summarizes read counts to clusters, ready for statistical testing. Using a range of metrics, we demonstrate that Corset out-performs alternative methods. Corset is available from https://code.google.com/p/corset-project/.

**Electronic supplementary material:**

The online version of this article (doi:10.1186/s13059-014-0410-6) contains supplementary material, which is available to authorized users.

## Background

Next-generation sequencing of RNA, RNA-seq, is a powerful technology for studying various aspects of the transcriptome; it has a broad range of applications, including gene discovery, detection of alternative splicing events, differential expression analysis, fusion detection and identification of variants such as SNPs and post-transcriptional editing [1,2]. One of the advantages of RNA-seq over older technology, such as microarrays, is that it enables the transcriptome-wide analysis of non-model organisms because a reference genome and annotation are not required for generating and analyzing the data. When no reference genome is available, the transcriptome is *de novo* assembled directly from RNA-seq reads [3]. Several programs exist for *de novo* transcriptome assembly: Oases [4] and Trans-abyss [5], which extend the Velvet [6] and Abyss [7] genomic assemblers, respectively, as well as purpose built transcriptome assemblers such as Trinity [8]. These programs are capable of assembling millions of short reads into transcript sequences - called contigs.

One common and biologically important application of RNA-seq is identifying genes that are differentially expressed between two or more conditions [9]. However, performing a differential expression analysis on a *de novo* assembled transcriptome is challenging because multiple contigs per gene are reported. Multiple contigs, with shared sequence, arise because transcriptome assemblers differentiate between isoforms of the same gene, and report each separately. Furthermore, they often report contigs that are not truly representative of different isoforms but arise from artifacts such as sequencing errors, repeats, variation in coverage or genetic variation within a diploid individual or pooled population. As a result, transcriptome assemblers often report fragmented versions of a transcript, or repeated contigs that differ only by a SNP or indel. Surprisingly, simulations have shown that even the assembly of data without any sequencing errors, SNPs or alternative splicing can generate multiple contigs per gene [10]. Hence, the number of contigs produced by a *de novo* assembly is typically large; for example, assemblies with 80 million reads can produce hundreds of thousands of contigs [11].

The inevitably long list of contigs generated by *de novo* transcriptome assembly causes several issues for differential expression analysis: i) reads cannot be aligned unambiguously to duplicated sequences and determining the origin of ambiguously aligned reads is error prone; ii) the statistical power of the test for differential expression is reduced as reads must be allocated amongst a greater number of contigs, thus reducing the average counts per contig; iii), the adjustment for multiple testing is more severe; and iv), once differentially expressed contigs have been identified, interpretation is difficult, as many genes will be present in the list multiple times. Performing a differential expression analysis on genes, rather than contigs, would overcome these difficulties. However, the procedure for estimating gene-level expression from a set of *de novo* assembled contigs is not straightforward and has not been thoroughly examined in the literature.

**Figure 1:**
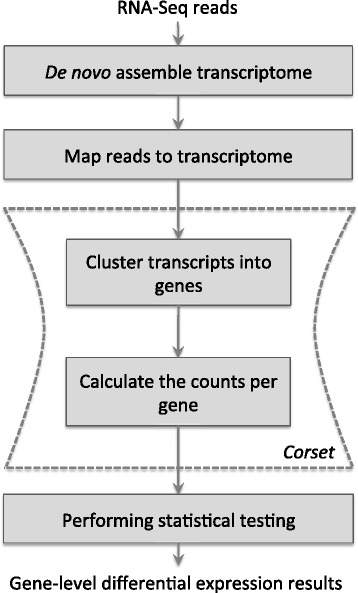
**The pipeline for performing a count-based gene-level differential expression analysis on non-model organisms.** Cleaned RNA-seq reads are first *de novo* assembled into contig sequences. Reads are mapped back to the transcriptome and the association between contigs and genes must be established (clustering of contigs). Then the abundance of each gene is estimated. Finally, statistical testing is performed on the count data to determine which genes are differentially expressed. Corset performs the clustering and counting (dashed box) in a single step.

Several steps are involved in identifying differentially expressed genes from a *de novo* assembled transcriptome (Figure 1): RNA-seq reads are first assembled, reads are next mapped back to contigs, contigs are then clustered into genes, after which the expression level for each gene cluster is summarized, and statistical testing is performed to detect differential expression.

Several studies have compared individual steps in this analysis pipeline. For example, the relative merits of different *de novo* assemblers and steps prior to assembly, such as quality control, have been examined [12–15]. Similarly, the choice of method for performing count-based statistical testing for differential expression has been evaluated [16,17]. However, few studies have compared or even suggested a path for obtaining gene-level counts from transcriptome assemblies [18,19] and only a single automated pipeline has thus far been implemented to address this need [20]; it is provided by Trinity to run RSEM [21] followed by edgeR [22] or DESeq [23]. This pipeline is inflexible, however, to the choice of assembler.

In this paper we present Corset, a method and software for obtaining gene-level counts from any *de novo* transcriptome assembly. Corset takes a set of reads that have been multi-mapped (multiple alignments per read are reported) to the *de novo* assembled transcriptome and hierarchically clusters the contigs based on the proportion of shared reads and expression patterns. Expression patterns allow for discrimination between genes that share sequence, such as paralogues, if the expression levels between groups are different. Using the mapped reads, Corset then outputs gene-level counts. The gene-level counts can then easily be tested for differential expression using count-based frameworks such as edgeR and DESeq. We demonstrate that Corset consistently performs well compared to alternative clustering methods on a range of metrics. Moreover, as it is an assembler-independent method, it allows contigs and transcripts from various sources to be combined. It is also simpler to use, with the clustering and counting steps encompassed in a single run of the software.

## Results and discussion

### Corset clusters contigs and counts reads

The first step in performing a gene-level differential expression analysis for a non-model organism is to assemble the contigs, which can be performed using a variety of software. As previously outlined, this process produces multiple sequences or contigs per gene. Consequently, the next step is to group, or cluster, the contigs into genes to facilitate downstream differential expression analysis. This clustering step is the first step of Corset.

Corset requires that, after transcriptome assembly, reads are mapped back to the contigs allowing reads to map to multiple contigs (multi-mapping). These multi-mapped reads are then used as a proxy for detecting sequence similarity between contigs, as well as providing information about the expression level of the contigs. Corset also uses the read information to filter out contigs with a low number of mapped read (less than 10 reads by default). Corset’s approach is in contrast to other tools used for clustering contigs as the majority of other tools only use the sequence information from the assembly.

Corset works by clustering contigs based on shared reads, but separates contigs when different expression patterns between samples are observed. This is implemented using an agglomerative hierarchical clustering algorithm. The distance between any two contigs is defined in relation to the number of reads that are shared between contigs, such that a lower proportion of shared reads results in a larger distance (see Materials and methods). Genes that share sequence, such as paralogues, are likely to have small distances, as many reads are shared. As we do not want these contigs to be clustered, Corset performs a test to detect whether the relative expression levels between the pair of contigs is constant across conditional groups (or experimental groups). If the relative expression between the two contigs is not constant, the distance between the two contigs is set to the maximum. This is incorporated into the algorithm as a likelihood ratio test where the null hypothesis assumes that the ratio between counts from the two contigs are equal across conditional groups, whereas the alternative hypothesis allows this ratio to vary with conditional group. The count data for this contig ratio test are modeled as Poisson distributed and a *P*-value threshold of approximately <10^-5^ is applied by default (see Materials and methods for a detailed description and justification of thresholds).

**Figure 2:**
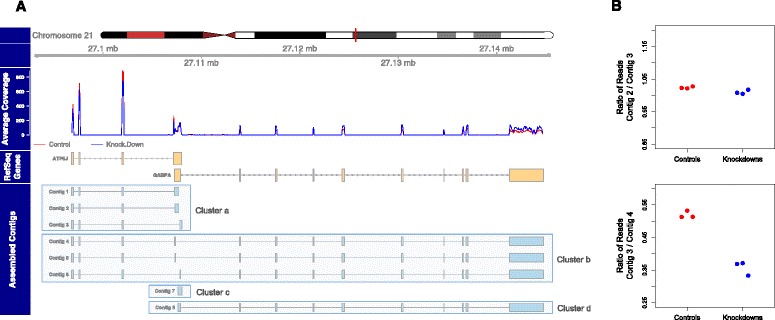
**Corset uses expression information to tease apart contigs from different genes. (A)** Assembled contigs from a region of the human genome containing the two genes *ATP5J* and *GABPA*. Trinity assembles 8 contigs (bottom track), which are grouped into one cluster if the contig ratio test is not applied. Including this test allows corset to separate this region into four clusters (boxes). Notably, contigs 4 to 6 are false chimeras, caused by the overlapping UTRs of *ATP5J* and *GABPA*. These genes are differentially expressed, as shown by base-level coverage, averaged over replicates (top track). **(B)** When clustering, Corset checks for equal expression ratios between conditions when calculating distances between pairs of contigs: here we consider pairs contigs 2 and 3 (top) and contigs 3 and 4 (bottom). The ratio of the number of reads aligning to each contig is plotted for each sample (dots). It can be seen that contig 2 and contig 3 have the same expression ratio across groups and so are clustered together while contig 3 and contig 4 have different expression ratios between conditions and so are split. This feature helps Corset separate contigs that share sequence but are from different genes.

The contig ratio test that separates contigs with shared sequence but differing expression ratios is one of the novel features of the Corset clustering algorithm. Although this feature can be switched off - for example, to ensure differentially spliced isoforms are clustered together - we find it is effective in separating contigs from different genes (Additional file 1: Figure S7). For example, Figure 2 shows the human *ATP5J* and *GABPA* genes, which reside on opposite strands but have overlapping UTRs. The assembly of human primary lung fibroblast data produced eight contigs for this region (see Materials and methods). While there are contigs for each of the genes separately (contigs 1 to 3, and 8) the use of a non-stranded protocol results in contigs with the two genes assembled together (contigs 4 to 6). When the contig ratio test is not implemented, all these contigs are assigned to the same cluster and no significant differential expression is detected between the knock-down and wild-type conditions (false discovery rate (FDR) = 0.053). However, examining the contig count ratios between pairs of contigs tells a different story (Figure 2B). The count ratios of contig 3 and contig 2 are constant across samples, implying they should be in the same cluster. By contrast, the contig ratio between contig 3 and contig 4 is significantly different across conditions and so Corset splits them into different clusters. When tests for all pairwise combinations are performed, these eight contigs are separated into four different clusters and statistical testing for differential expression reveals cluster a and d are significantly differentially expressed in opposite directions (FDR = 10^-11^ and 10^-7^, respectively).

Once Corset is applied to the full dataset the contig groupings that are representative of genes are reported and will be referred to henceforth as clusters. Corset also reports the number of read counts associated with each cluster. All the reads are uniquely assigned to a cluster (see Materials and methods); hence, each read is only counted once, even though the reads were originally multi-mapped to contigs. The read counts table can be supplied to count-based differential expression programs for statistical testing.

### Testing Corset on model organism datasets

We tested the performance of Corset against other clustering and counting methods using three RNA-seq datasets: chicken male and female embryonic tissue [24], human primary lung fibroblasts, with and without a small interfering RNA (siRNA) knock down of *HOXA1* [25], and yeast grown under batch and chemostat conditions [26]. We selected three model organisms in order to compare our *de novo* differential gene expression (DGE) results against a genome-based analysis (referred to herein as the truth dataset). In the chicken dataset we tested for DGE between males and females. The homology between chicken genes, which is around 90% on the sex chromosomes [24], offered a challenging test for clustering algorithms. The human dataset was selected because human is one of the best annotated species and the yeast was used to assess whether clustering is beneficial for organisms with minimal splicing. Each dataset was assembled using Trinity and Oases, which have different underlying assembly strategies, to ensure that the results were consistent. Overall, six different assemblies were used as a starting point for the evaluation of Corset.

### Corset clustering results in a good balance between precision and recall

We were initially interested in comparing the clustering produced by Corset with other available methods. Both Trinity and Oases provide some clustering information with their output, which is based on the partitioning of the de Bruijn graphs during the assembly (referred to as components and locus, respectively). Standalone tools based on sequence similarity are also frequently used [27,28], with CD-HIT-EST a popular choice [29,30].

We evaluated Corset’s clustering against CD-HIT-EST and the assemblers’ own clustering. For chicken, over 300,000 contigs were assembled while for human, over 100,000 contigs were assembled (Table 1). A large number of clusters were reported by Trinity and CD-HIT-EST - for example, over 200,000 clusters on the chicken dataset. By default, Corset removes contigs with a very low number of reads supporting them, to give fewer clusters in all cases (Additional file 1: Table S1). This makes the cluster list more manageable, without compromising sensitivity to detect differential expression. Oases also gave fewer clusters than CD-HIT-EST, but it grouped many unrelated contigs together, with the largest clusters containing many thousands of contigs (Table 1).

**Table 1:** **Statistics on the number of clusters for various clustering options compared to Corset**

		**Chicken**		**Human**		**Yeast**	
		**Trinity**	**Oases**	**Trinity**	**Oases**	**Trinity**	**Oases**
**Contigs**		**335,377**	**540,933**	**107,389**	**239,426**	**7,353**	**27,013**
Trinity	Clusters (Max.)	230,924 (302)		73,258 (91)		6,690 (45)	
Oases	Clusters (Max.)		87,639 (93,103)		55,746 (16,881)		3,140 (5,987)
CD-HIT-EST	Clusters (Max.)	282,285 (81)	202,636 (116)	90,115 (29)	96,965 (74)	7,117 (8)	5,586 (39)
Corset	Clusters (Max.)	91,653 (290)	67,826 (208)	43,663 (90)	38,476 (59)	3,796 (45)	4,324 (65)

Shown are the number of contigs (bold), number of clusters and the maximum number of contigs in a cluster (in parentheses). Corset removes contigs that have less than 10 reads mapping to them by default, and hence has the least number of clusters in 5 out of 6 assemblies. This makes the final list of clusters more manageable, with no detriment to the final DGE results. Oases grossly over-clusters as shown by the maximum contigs in a cluster.

Clustering was evaluated using precision (True positives/(True positives + False positives)) and recall (True positives/(True positives + False negatives)) for each of the six *de novo* assemblies. Positives and negatives were calculated by taking all pairwise combinations of contigs and evaluating if the contigs were correctly placed in the same cluster (true positives), correctly separated into different clusters (true negatives), incorrectly placed in the same cluster (false positives) or incorrectly separated (false negatives) [31]. Truth information was derived using the appropriate reference genome annotation (see Materials and methods). Contigs filtered out by Corset due to a low number of mapped reads were also filtered out for the assessment of competing methods.

**Figure 3:**
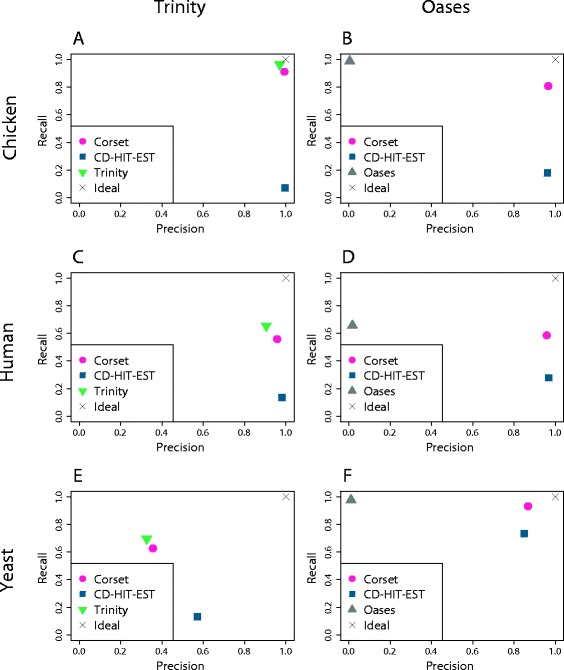
**A comparison of the performance of different clustering approaches.** For the assembler’s own clustering (Trinity or Oases), CD-HIT-EST and Corset we show the precision against the recall. The precision is the ratio of true positives over true positives plus false positives and the recall is the ratio of true positives over true positives plus false negatives. We show the results for six different assemblies: **(A)** chicken data assembled with Trinity; **(B)** chicken data assembled with Oases; **(C)** human data assembled with Trinity; **(D)** human data assembled with Oases; **(E)** yeast data assembled with Trinity; and **(F)** yeast data assembled with Oases. The X indicates perfect clustering.

We found that CD-HIT-EST was generally high in precision but poorer in recall. In contrast, Oases’ clustering performed well in recall but had a precision around zero in all cases. Conceptually, this again indicates that Oases groups many unrelated contigs into the same cluster (over-clustering). The clustering from Trinity showed a better balance between precision and recall. Corset outperformed both CD-HIT-EST in recall and the assembler’s clustering in precision in all cases (Figure 3), indicating that it provides a good balance between precision and recall. In addition, in two out of the six assemblies, Corset was the most precise (chicken-Oases and yeast-Oases).

Clustering performance is influenced by not only the choice of clustering algorithms but also the choice of assembler and the quality of the RNA-seq dataset. We briefly investigated how certain aspects of the assembly quality affect clustering recall and precision. We found that recall decreases with greater fragmentation of genes in the assembly. Contigs from a common gene that share no sequence are unlikely to be clustered together by any algorithm, whereas contigs that are almost fully redundant should always be clustered together. The majority of genes fall into one of these two extremes (Additional file 1: Figure S2B). The assemblers’ clustering and Corset behaved as expected, giving close to perfect recall for genes with fully overlapping contig sequence and zero recall when the contigs were disjointed. However, CD-HIT-EST failed to achieve good recall even for genes with no fragmentation (Additional file 1: Figure S4). The fraction of fully disjointed contigs appears to dictate an upper bound on the best possible recall that can be achieved by any clustering algorithm.

We found that poor clustering precision, whereby contigs from different genes are grouped together, happens when genes share sequence, such as paralogues, a common domain, overlapping UTRs or repeats. In some cases, this can also result in a chimeric contig being erroneously assembled (for example, Figure 2). It has previously been illustrated that there is a high rate of false chimeras in *de novo* transcriptome assemblies [32] and we also observed a high rate of false chimeras in our assemblies, around 5 to 15% of contigs for chicken and human, and 40% of contigs for yeast (Additional file 1: Figure S3). Oases’ clustering precision on genes that share sequence or have chimeric contigs was consistently worse than that of Corset and CD-HIT-EST. Trinity was marginally worse than Corset. For genes that had no shared sequence, perfect clustering precision was seen for Corset and CD-HIT-EST (Additional file 1: Figure S5).

These results indicate that clustering performance is influenced by the underlying assembly quality (which in turn depends on the dataset), but that Corset clustering is robust over a range of assembly qualities.

### The effect of clustering on differential gene expression results

**Figure 4:**
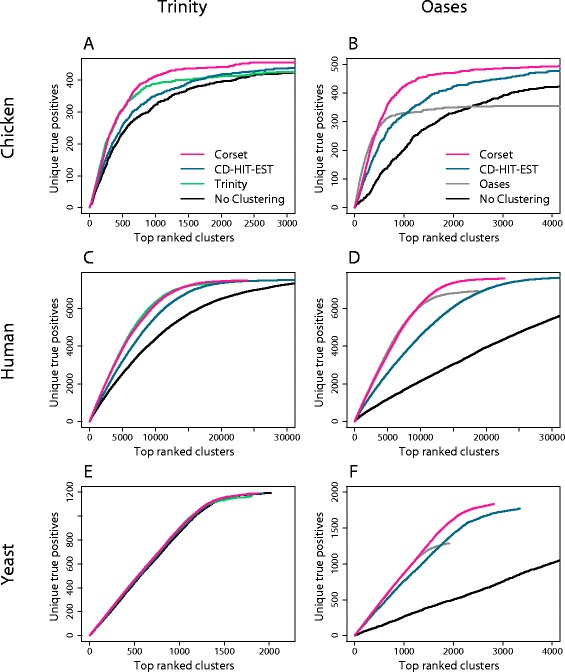
**The effect of clustering on differential gene expression rankings.** The cumulative number of unique true positive differentially expressed clusters against the number of top ranked clusters in the *de novo* analysis is shown. A unique true positive refers to only counting the first instance of a gene that appears multiple times in the ranked list. Corset performed the same or better than CD-HIT-EST and the assembler’s own clustering, in all cases: **(A)** chicken data assembled with Trinity; **(B)** chicken data assembled with Oases; **(C)** human data assembled with Trinity; **(D)** human data assembled with Oases; **(E)** yeast data assembled with Trinity; and **(F)** yeast data assembled with Oases. For comparison, we also show the results of no clustering, where the analysis was performed at the level of contigs rather than clusters.

Poor precision is akin to over-clustering, making some differentially expressed genes impossible to detect because contigs with different relative expressions are combined. Moreover, the functional annotation of clusters becomes ambiguous. Poor recall, however, is akin to under-clustering. It extends the total length of the list of clusters, which has several consequences: it is inconvenient for follow-on studies (such as gene ontology), leads to greater multiple testing corrections and increases statistical uncertainty. To assess the extent of these effects on differential expression results, we performed a gene-level differential expression analysis using each of the clustering options. The remaining steps in the pipeline, including count-based abundance estimation, were identical in each case (see Materials and methods) and testing for DGE was performed using edgeR. Significantly differentially expressed clusters were compared to genes tested for differential expression using a genome-based mapping approach. A cluster was deemed to be a true positive if it matched a differentially expressed gene from the genome-based analysis. Regardless of the statistical test used to generate true differentially expressed genes from the genome based analysis, Cuffdiff 2 [25] (Figure 4) or edgeR (Additional file 1: Figure S10), we found similar results in the comparison of Corset to other contig clustering options.

**Figure 5:**
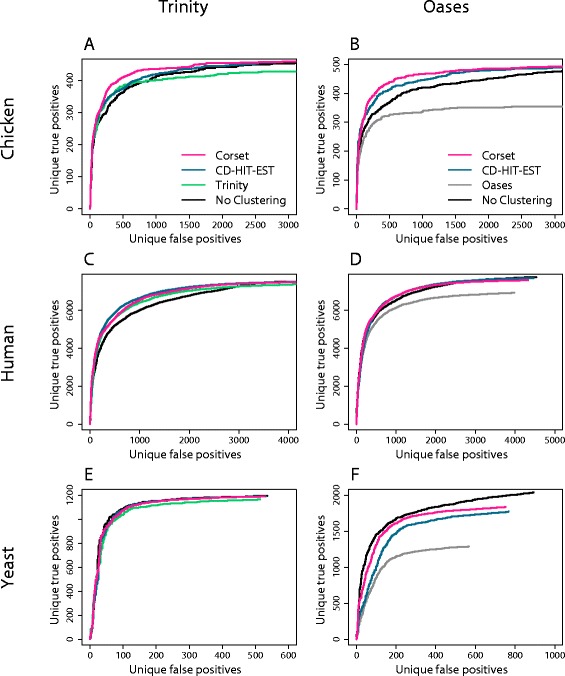
**The effect of clustering on differential gene expression receiver operating characteristic (ROC) curves.** The unique true positive differentially expressed clusters against unique false positive clusters in the *de novo* analysis is shown. A unique positive refers to only counting the first instance of a gene that appears multiple times in the ranked list. Corset performed similarly to or better than CD-HIT-EST and the assembler’s own clustering, in all cases: **(A)** chicken data assembled with Trinity; **(B)** chicken data assembled with Oases; **(C)** human data assembled with Trinity; **(D)** human data assembled with Oases; **(E)** yeast data assembled with Trinity; and **(F)** yeast data assembled with Oases. For comparison, we also show the results of no clustering, where the analysis was performed at the level of contigs rather than clusters.

We looked at three different measures to assess the impact of clustering on DGE results. Firstly, we examined the cumulative number of unique true positive clusters as a function of the total number of clusters (Figure 4). A unique true positive refers to only counting the top ranked cluster when there is more than one cluster assigned to a gene. In four cases Corset performed better than the alternatives (chicken-Trinity, chicken-Oases, human-Oases, yeast-Oases) and in the remaining two cases it performed equally well (human-Trinity, yeast-Trinity). This metric penalized for reporting multiple clusters for a given gene (that is, poor recall). It was also informative to examine an alternative version of this metric that does not penalize in this way: the number of unique true positives as a function of the number of unique false positives (Figure 5). In this instance, clustering algorithms with better precision do better than the assembler’s clustering, which performed better in recall. As a final assessment of clustering we looked at the correlation in fold change between differentially expressed genes from the truth analysis, and those from the *de novo* assembly (Table 2). Corset was consistently the most concordant with the genome-based truth analysis.

**Table 2:** **Pearson correlation in gene-level log**_**2**_ **fold changes**

	**Chicken**		**Human**		**Yeast**	
	**Trinity**	**Oases**	**Trinity**	**Oases**	**Trinity**	**Oases**
No clustering	0.720	0.734	0.884	0.835	**0.968**	0.958
Trinity	0.820		0.933		0.934	
Oases		0.447		0.888		0.760
CD-HIT-EST	0.751	0.756	0.919	0.929	**0.968**	0.903
Corset	**0.874**	**0.850**	**0.936**	**0.956**	**0.968**	**0.974**

In previous validation results, we assessed clustering by examining the ranking of true positives. Here we assess how well the fold change between experimental conditions is recovered. For each contig matching a gene with true differential expression, we compared its cluster-level log2 fold change against its true gene-level log2 fold change. The Pearson correlation between these quantities is shown. We assessed each clustering method in this way and found corset clustering gave the highest correlation in all cases. The highest Pearson correlation for each assembly is displayed in bold.

The DGE results also illustrate the general importance of clustering contigs into genes; the differential expression analysis on contigs with no clustering resulted in a much longer list for the same number of unique true positives compared to clustering (Figure 4). This was even the case for the Oases assembly from yeast, an organism with little alternative splicing (Figure 4F), highlighting the importance of removing redundancy from the assembly, even for genomes where minimal alternative splicing is expected. By all metrics, Corset was the best or close to best method available. This indicates that the balance between precision and recall that Corset achieves translates into more accurate DGE results.

### Corset allows multiple transcriptomes to be combined

An ideal clustering tool would allow transcriptomes generated from different sources to be combined because multiple transcripts from the same gene will be clustered together regardless of their origin. However, this is only possible for clustering that is independent of the *de novo* assembler. While several publications have used CD-HIT-EST for combining multiple transcriptome assemblies [12,14,29], we have already shown that CD-HIT-EST is not the most effective contig clustering tool. Corset, however, provides a convenient method to cluster contigs generated from different sources. Reads are first multi-mapped to each transcriptome separately, and then all bam files are processed together in one run of Corset.

Different assemblers have strengths and weaknesses and it is often advantageous to combine the results from several *de novo* assemblers. To demonstrate the utility of Corset for this purpose we clustered together the Trinity and Oases assemblies from the human dataset. This combined dataset effectively doubled the number of contigs. Corset was able to handle this level of redundancy to give a combined transcriptome with fewer clusters, 37,741, than either of the Trinity or Oases assemblies individually, 43,664 and 38,477, respectively. Furthermore, this combined transcriptome contained contigs annotating approximately 200 additional genes not detected using either constituent transcriptome alone. By contrast CD-HIT-EST produced 115,980 clusters on the same combined dataset.

**Figure 6:**
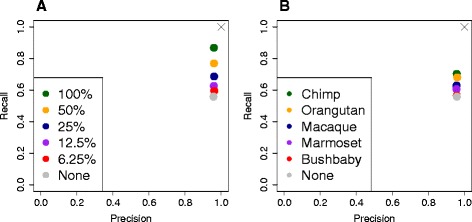
**Supplementing a** ***de novo*** **assembly with additional transcriptomes.** Supplementing a *de novo* assembly with contigs from either **(A)** a partial annotation or **(B)** related species improves clustering recall of the *de novo* assembled contigs. We show the recall and precision, calculated for Trinity contigs. **(A)** We randomly sampled transcripts from the human annotation from Ensembl at 100%, 50%, 25%, 12.5% and 6% of all transcripts to emulate a partial annotation, mapped the human RNA-seq reads to each set and clustered the reads together with those mapped to the Trinity assembly using Corset. **(B)** We mapped human RNA-seq reads onto the Ensembl annotation for chimp, orangutan, macaque, marmoset and bushbaby, then clustered the reads together with those mapped to the Trinity assembly using Corset. 'None' in both plots indicates the Trinity assembly on its own.

Another application for combining transcriptomes is when a partially assembled genome or annotation is available. Supplementing *de novo* assembled data with genome-based data has several advantages: i) it increases the amount of known transcript sequence, for example, because genes, or regions of genes in the annotation, that have little or no read coverage are absent from the assembly; ii) *de novo* assembled contigs can be easily annotated if they cluster with a known gene; and iii) it allows disconnected fragments in the assembly to be clustered together if a transcript from the reference annotation overlaps both. We demonstrated this final benefit by combining the Trinity transcriptome from the human RNA-seq dataset with the human Ensembl version 73 annotation using Corset. We randomly sampled 50%, 25%, 12% and 6% (approximately 100, 50, 25 and 12.5 thousand transcripts) of the full Ensembl transcriptome to emulate a partial annotation. A significant improvement in clustering recall is seen for Trinity contigs with no detriment to clustering precision (Figure 6A).

Finally, we extended this idea to the use of a reference annotation from a related species [33]. The human RNA-seq reads were mapped independently to the Trinity assembly and Ensembl version 73 transcript sequences for chimp, orangutan, macaque, marmoset and bushbaby, with lower mapping stringency used for the related species (see Materials and methods). Our results demonstrate that an improvement in clustering recall is obtained even using annotation from a related species, again without loss of precision (Figure 6B). The effectiveness of this strategy will depend on the divergence between species, which in this example ranged from approximately 6 million years ago (My; chimp), 15 My (orangutan), 32 My (macaque), 46 My (marmoset) to 68 My (bushbaby) [34]. In addition, the improvement will also depend on the completeness of the annotation (these species had half to one-third as many transcripts as the human Ensembl annotation).

### Corset summarizes reads into gene-level counts

As previously mentioned, Corset not only clusters contigs but also produces expression levels for each cluster, in the form of read counts that can be directly used by DGE analysis software. This feature is provided as a convenience because it replaces the two-step process of clustering contigs and estimating abundances with a single step.

We compared the performance of the counting aspect of our software against three other pipelines for gene-level count estimations: (1) RSEM [21]; (2) selecting the longest contig from each cluster as a representative sequence and mapping to that; and (3) mapping each read to all contigs, allowing only a single alignment, then aggregating the counts within a cluster (see Materials and methods). In each case, the Corset clustering was used. We found that, in general, all counting methods produced similar results to Corset; the Pearson correlation between counts produced by Corset and other methods was consistently high (Additional file 1: Table S2). Despite RSEM and Corset reporting identical counts for up to 95% of clusters (Additional file 1: Table S3), we found a significant difference in counts for a small number of clusters. In these clusters RSEM tended to report fewer counts (Additional file 1: Figure S11A). Furthermore, for these clusters Corset counts exhibited less variability between biological replicates (Additional file 1: Table S4 and Figure S11B), suggesting that they were more precise. As a final assessment of the discrepancy, we compared RSEM cluster-level counts against a truth set where the truth was constructed by running RSEM on reads mapped to the gene annotation (Additional file 1: Figure S12A). This confirmed that RSEM was underestimating the counts for a small proportion of clusters, resulting in missing true differential expression in the downstream analysis (Additional file 1: Figure S12B).

Hence, we found no evidence that there was a disadvantage in using the counts produced by Corset over other programs, such as RSEM, for gene-level analysis. On the contrary, we found subtle hints that simple count-based abundance estimation methods may be more robust for detecting differential expression on *de novo* assembled transcriptomes than methods that attempt to infer abundance at the transcript level first. However, we reiterate that all counting methods gave similar results.

## Conclusions

Recently, there has been a focus on the potential for using RNA-seq to analyze the transcriptomes of non-model organisms, with a number of studies exploring various steps in the analysis pipeline, such as the effect of cleaning reads [13], digital normalization [35], different assemblers [12] and post-assembly improvements [36]. However, in many applications of RNA-seq the outcome of interest is not the transcriptome itself, but the detection of differentially expressed genes between samples. To this end, there are few tools or even guidelines on how to progress from the assembled transcriptome to a list of differentially expressed genes. This study presents a novel algorithm, Corset, for clustering *de novo* assembled contigs and generating gene-level counts. This study is also the first to compare various pipelines for DGE analysis of *de novo* assembled transciptomes, demonstrating that it is advantageous to perform a gene-level rather than transcript-level analysis, even on species such as yeast, with minimal alternative splicing.

However, performing a gene-level analysis requires the *de novo* contigs to be clustered into genes. Prior to the algorithm presented here, clustering options were limited to either the assembler’s own groupings or a sequence-based clustering tool. Furthermore, it was not clear how well these methods performed in the context of a *de novo* assembled transcriptome; specifically, their ability to deal with issues such as the fragmentation of genes, erroneous chimeras and repeats, all of which may lead to the miss-clustering of contigs.

Our method, Corset, makes use of both the sequence similarity and expression data available to cluster contigs. The use of expression data means Corset has the power to separate paralogues and contigs with erroneous chimeras into different clusters. A possible consequence of separating contigs based on relative expression is that differentially spliced isoforms of a gene may be split into separate clusters. For gene-level differential expression analysis, we see no disadvantage in this; either or both isoform clusters should be detected as differentially expressed. However, this option can be turned off should the user want to ensure isoforms are clustered together. Overall, we found the clustering provided by Corset performed better than alternative approaches in several metrics we examined. Similarly, the expression data provided as counts by Corset gave results equal to, and sometimes marginally more accurate than, all alternative estimates.

Thus, Corset provides new methods in a single software tool that effectively replaces the often ambiguous, cumbersome, multi-step process required to go from a *de novo* assembled transcriptome to gene-level counts. Corset is easy to run as no indexing or sorting of the bam files is required and it can process single-end, paired-end or mixed reads. Finally, Corset provides a convenient way to merge the results from different *de novo* assemblies, reference annotations or genome-guided assemblies. We believe these features will be of great benefit to RNA-seq analysis in non-model organisms.

## Materials and methods

### Datasets

We performed differential gene expression analysis using publicly available RNA-seq data from three model organisms: chicken, human and yeast. All datasets consisted of 100-bp paired-end reads from an Illumina HiSeq 2000. For each dataset we trimmed the reads [37] and then performed three analyses: two on *de novo* transcriptomes assembled using Oases and Trinity and one genome-based analysis - the 'truth' - which was used for comparison. The chicken dataset from Ayers *et al*. [24], Short Read Archive (SRA) accession number SRA055442, consisted of approximately 1.2 billion reads. For the *de novo* analyses we used only one lane of this data (approximately 320 million reads) because the full dataset was computationally too large to assemble. However, all the data were used for the genome-based 'truth' analysis. This dataset consists of eight samples - male and female blastoderms, and male and female day 4.5 gonad tissue, in duplicate. The dataset published by Trapnell *et al*. [25], Gene Expression Omnibus accession GSE37704, is from human primary lung fibroblasts with an siRNA knock-down of *HOXA1*. The dataset contains three replicates of the knockdown and three controls with more than 231 million reads in total. Finally, we included a yeast dataset, SRA accession numbers SRR453566 to SRR453571, published in Nookaew *et al*. [26]. The dataset consists of approximately 36 million reads. Three replicates were grown under batch conditions and three under chemostat conditions.

### Genome-based 'truth' analysis

To gauge the performance of different clustering and abundance estimation algorithms, we derived a 'truth' set using genome-based analysis.

To determine the correspondence between *de novo* assembled contigs and reference annotation genes, we aligned the assembled contigs against the annotation using BLAT [38] (minimum length of 200 bases and minimum identity of 98%). Chimeric contigs were treated as having an unknown origin. We identified chimeric contigs as those that matched two or more truth genes (as above) with an overlap between the genes of less than 100 bases. For other cases where a contig aligned to multiple genes, it was assigned to the gene with the longest alignment length. When comparing the differentially expressed 'truth' genes to *de novo* clusters, we assigned a cluster to the same 'truth' gene as the majority of its contigs. Any contig or cluster that could not be found in the 'truth' set was excluded from the results shown. Contigs that were removed by Corset due to a low number of reads mapping were also excluded.

To calculate 'true' differential expression, reads were first mapped using TopHat v2.0.6 [39] to either the hg19, galGal3 or sacCer3 versions of the human, chicken and yeast genomes, respectively. In all cases we provided the gene annotation (RefSeq for human, Ensembl (v.70) for chicken and Saccharomyces Genome Database for yeast) to TopHat to support splice site detection. These same gene annotations were processed by 'gffread --merge' to give locus level annotations. Cuffdiff 2.1.1 was run to detect differential gene expression (with the -*u* option). We used 'significant' locus in 'gene_exp.diff' as true positives. As an alternative to cuffdiff 2 we also defined truth using a genome based edgeR analysis (results shown in Additional file 1: Figure S10). EdgeR was run in the same way as for the *de novo* assembly (see 'Statistical testing' below).

### *De novo* assembly

Oases 0.2.06 (with Velvet version 1.2.07) was used to assemble the human and yeast data with kmer lengths of 19, 23, 27 and 31. For the chicken dataset we used kmer lengths of 31, 41, 51, 61 and 71. The chicken Trinity assembly was created using Trinity-r2012-10-05 and the human and yeast assemblies using Trinity-r2013-02-25. Default parameters were used in all cases, with a minimum contig length of 200 bases. Additional file 1: Figures S1, S2 and S3 show the assembly quality.

### Mapping

Reads were mapped to the *de novo* assemblies as paired-end alignments using bowtie [40]. For single-mapping, where only one alignment was allowed, we used the bowtie option *--best*. For multi-mapped alignment, we used the option *--all*. When mapping to related species we used the bowtie settings, -*-all -m 6 -n 3 -e 1000 -X 1000*, to allow for a great number of mismatches. For the human dataset, this resulted in between 30% (bushbaby) and 70% (chimp) of read pairs mapping, compared to about 75% for the Trinity assembly.

### Clustering

We clustered the transcriptomes using CD-HIT-EST with default parameters. For the assembler clustering, we extracted the clusters from the contig names in the assembly fasta file. For example, for Trinity, the contig 'comp1_c2_seq3' belonged to the cluster 'comp1_c2'. For Oases, 'Locus_1_Transcript_3/10_Confidence_0.000_Length_268' belonged to cluster 'Locus_1'. To obtain the Corset clustering we multi-mapped the reads to the transcriptome and executed Corset with the experimental groups included as a parameter (-*g* option). For the differential expression results presented in Figures 4 and 5 and Table 2, we estimated the counts using the 'single-mapping then summation' method described below.

### Abundance estimation analysis

The four methods described below were compared to assess which gave the best DGE results. In all cases the clustering was identical and was generated using Corset with the experimental groups passed through the -*g* options and using the -*m 0* option (so that all contigs were reported). The statistical testing was performed using edgeR.

#### RSEM

Multi-mapped bam files were converted to the format required by RSEM using the command, 'convert-sam-for-rsem'. The transcriptome was prepared using 'rsem-prepare-reference --no-polyA --no-bowtie --transcript-to-gene-map' with Corset clustering passed as a parameter. The gene abundance was estimated using 'rsem-calculate-expression --bam --paired-end' and the 'expected_counts' were extracted from the '.genes.results' files.

#### Representative contig method

The longest contig was selected to represent each cluster. Reads were single-mapped back to these contigs. The number of reads mapping to each representative contig was counted using the samtools idxstats command. Because these count data were per read, we divided by two to get the counts per fragment.

#### Single-mapping then summation

We single-mapped reads to all contigs and counted the number overlapping each using samtools idxstats [41]. To obtain gene-level counts, we summed the counts for all contigs within a cluster. Because these count data were per read, we divided by two to get the counts per fragment.

#### Corset

We multi-mapped the reads to the transcriptome and executed Corset with the options described above.

### Statistical testing

The cluster-level count data were processed using edgeR. For the chicken data, we modeled the data with four conditional groups (two sex and two time-points) as in Ayers *at al*. [24], but tested for a difference between males and females from the later time-point only. The other datasets had two conditional groups each (with three replicates for a total of six samples) and the statistical testing was performed for differences between these groups. We used the edgeR GLM framework in all cases with tagwise dispersion estimation [42]. The statistical testing was performed in the same way for all *de novo* assemblies. Statistical testing for the 'truth' genome-based analysis was done using Cuffdiff 2 (Figures 4 and 5) and edgeR (Additional file 1: Figure S10). While these gave a slightly different list of significant truth genes, the results comparing Corset to alternative clustering methods were similar.

### The Corset algorithm

Our software accepts a set of multi-mapped read alignments in bam format (one or more files per sample) as input. The algorithm then proceeds in the following way:

1. Each read alignment is parsed and the read and contig IDs are extracted. For each read we store the set of contigs that it maps to.
2. Contigs with 10 or fewer reads are filtered out. This step is not essential to the algorithm, but has the effect of reducing the final total number of clusters as well as the average number of contigs per clusters, which can simplify the subsequent steps in the analysis.
3. The read data are parsed and super clusters are formed. Each super cluster contains all contigs that share one or more reads with another contig in the same super cluster.
4. Then for each super cluster we perform agglomerative hierarchical clustering similar to the algorithm in [43], but with distance and linkage described below. Hierarchical clustering is used rather than other clustering approaches because it is computationally tractable.

4.1 We create a distance matrix using the metric:

$$ distance = \left\{\begin{array}{c}\hfill 1-\frac{R_{a b}}{ \min \left({R}_a,{R}_b\right)}, contig\  ratio\  the\  same\hfill \\ {}\hfill 1, contig\  ratio\  different\hfill \end{array}\right. $$

where, *R*_*a*_ is the total number of reads that map to contig *a* across all samples, and *R*_*ab*_ is the total number of reads that map to both contig *a* and contig *b*, across all samples. The distance is therefore bounded between zero and one, with zero indicating a pair of redundant contigs and one indicating no similarity. 'Contig ratio' refers to the expression of contigs *a* and *b* being proportional to each other as measured across conditional groups. We make the assumption that this is true when two contigs originate from the same gene, and there is no alternative splicing. Alternatively, if the contigs do not come from the same gene or if there is alternative splicing, then their expression is not necessarily proportional, as can happen if one contig is differentially expressed. We test these scenarios using the 'contig ratio test', which proceeds in the following way. Let *r*_*aij*_ be the number of reads that map to contig *a* under condition *i*, for the *j*th replicate. We then approximate the number of reads that map to contig *a*, under condition *i* as:

$$ {X}_{ai}=1+{\displaystyle \sum_j}\left({r}_{ai j}+0.5{r}_{abij}\right) $$

The shared reads term, *r*_*abij*_, is used here to avoid double counting of reads. One is added as an offset to ensure that *X* > 0.

The contig-wise counts are then modeled as Poisson distributed. Note that we used a Poisson model for computational speed:

$$ {X}_{ai} \sim \kern0.5em \mathrm{Pois}\left({\mu}_{ai} = {f}_i{\mu}_{bi}\right) $$

$$ {X}_{bi} \sim \kern0.5em \mathrm{Pois}\left({\mu}_{bi}\right), $$

where *μ*_*ai*_ is the mean count for contig *a* under condition *i* and *f* is a proportionality constant. Define *f*_*i =*_*μ*_*ai/*_*μ*_*bi*_ as the true measure of proportional expression between contig *a* and *b* under condition *i.* We want to test the null hypothesis, *H*_*0*_*: f*_*i*_ *= f*_*i’*_ *= f,* that the proportionality constant is independent of condition, against the alternative, *H*_*1*_*: f*_*i*_ ≠ *f*_*i’*_.

Estimates of the proportionality constants for condition *i* are obtained from the contig-wise counts, that is:

$$ {\widehat{f}}_i=\frac{X_{ai}}{X_{bi}} $$

and the common proportionality constant is estimated by:

$$ \widehat{f}=\frac{{\displaystyle {\sum}_i}{X}_{ai}}{{\displaystyle {\sum}_i}{X}_{bi}} $$

We can test the null hypothesis using a likelihood ratio test with test statistic:

$$ \mathrm{D} = -2\left( \ln {l}_0- \ln {l}_1\right), $$

which is approximately chi-square distributed on *n*_*conditions*_ - 1 degrees of freedom under the null hypothesis. Here *n*_*conditions*_ is the total number of conditions, *l*_*0*_ is the likelihood under the null hypothesis and *l*_*1*_ is the likelihood under the alternative hypothesis.

Any pair of contigs for which the null hypothesis is rejected is defined as having a 'contig ratio difference' and will have its distance increased to the maximum value of 1. We found it convenient in terms of computation time to set a threshold on D that is equivalent to a *P*-value threshold of 10^-5^. The relationship between threshold and number of conditions is parameterized as *D*_*threshold*_ = 15 + 2.5 × *n*_*conditions*_. This relationship is only approximate and is valid when *n*_*conditions*_ < 10. This approximation should not affect the clustering, as we found the DGE results to be robust over a wide range of *P*-values (Additional file 1: Figure S7).

4.2 The hierarchical clustering proceeds by merging the two contigs with the smallest distance together. The number of reads that align to this new cluster is then updated, using the linkage criterion below, and the distance matrix is recalculated (as in step 3). Note that the linkage used by Corset differs from standard linkage approaches, such as single linkage, because it relies on information outside the distance matrix:

$$ {R}_{a'} = {R}_a + {R}_b\hbox{--} {R}_{a b} $$

$$ {R}_{a' c} = {R}_{a c} + {R}_{bc}-{R}_{a bc} $$

where contigs *a* and *b* are those being merged into cluster *a’. R*_*abc*_ is the number of reads mapping to all of contigs *a*, *b*, and *c*.

4.3 Steps 4.1 and 4.2 are iteratively repeated until either all the contigs have been grouped into a single cluster or the current minimum distance increases over the distance threshold. The clustering and number of reads per cluster is then output. Reads that align to multiple clusters are randomly assigned to one of the groups they align to. This accounted for only 1 to 5% of the 100-bp paired-end reads in our tests.

Our results were robust against the choice of distance threshold. The default value of 0.3 was chosen empirically because it was subtly better for DGE results (Additional file 1: Figure S9), but did not give significantly different results from any threshold between 0.1 and 0.9 (Additional file 1: Figures S8 and S9). The robustness with respect to threshold can be explain by most contigs pairs having a distance close to either 0 or 1 (for example, Additional file 1: Figure S2B).

The default *P*-value threshold for the likelihood ratio test, 10^-5^, was selected to account for the high level of multiple testing. This value was designed around the number of genes expected in a typical annotation. Again, we found that our results were robust against the choice of this parameter over a wide range, 10^-3^ to 10^-8^ (Additional file 1: Figure S7).

Our software is open source and is available as a C++ source code tar ball from [44]. It has been compiled and tested on Linux x86 and Mac OS X 10.7 operating systems. The duration of time needed for the code to complete varied from 5 minutes to 5 hours using one core of an Intel Xeon E7-8837 and was generally faster than the alternative pipelines. Memory consumption was less than 60 GB in the worst case, where over 200 GB of bam files were parsed by the program. The memory requirements were higher than other clustering and abundance estimation tools, but considerably less than the requirements for *de novo* assembly of the datasets we tested.

## Additional file

Additional file 1:
**Supplementary figures and tables.**

## Abbreviations

bp: base pair
DGE: differential gene expression
FDR: false discovery rate
My: million years ago
ROC: receiver operating characteristic
siRNA: small interfering RNA
SNP: single-nucleotide polymorphism
UTR: untranslated region
