# Supplementary material for: Corset: enabling differential gene expression analysis for de novo assembled transcriptomes
Source: Genome Biol. 2014 Jul 26;15(7):410. doi: 10.1186/s13059-014-0410-6 (PMC4165373; doi:10.1186/s13059-014-0410-6)
Supplement: Additional file 1: — Supplementary figures and tables. [file 13059_2014_410_MOESM1_ESM.docx]

Supplementary Figures and Tables for:

Corset: enabling differential gene expression analysis for *de novo* assembled transcriptomes

Nadia M. Davidson and Alicia Oshlack

Murdoch Childrens Research Institute, Royal Children’s Hospital, Flemington Road, Parkville 3052 Melbourne, VIC, Australia

Corresponding author: alicia.oshlack@mcri.edu.au

Contents:

Section 1: Validation of *de novo* assembly quality 2

Supplementary Figure 1

Supplementary Figure 2

Supplementary Figure 3

Section 2: Impact of *de novo* assembly quality on clustering 7

Supplementary Figure 4

Supplementary Figure 5

Supplementary Figure 6

Section 3: Supplementary data on Corset’s clustering 13

Supplementary Table 1

Supplementary Figure 7

Supplementary Figure 8

Supplementary Figure 9

Supplementary Figure 10

Section 4: Results for abundance estimation 18

Supplementary Table 2

Supplementary Table 3

Supplementary Figure 11

Supplementary Table 4

Supplementary Figure 12

Supplementary Table 5

**Section 1: Validation of *de novo* assembly quality**

**Supplementary Figure 1: The fraction of each gene assembled by any single contig.**

For each gene in the reference annotation we examined the maximum fraction of the gene sequence that was assembled by any single contig. Assembled contigs were matched to genes using BLAT (200 bases with 98% identity). Each point in the scatter plot is one gene. The blue line shows the median fraction recovered at a given expression quantile and the shaded area shows the 25%-75% quantile range. Trinity and Oases gave similar results. The yeast dataset had the highest fraction of genes assembled to full length, followed by chicken.

**
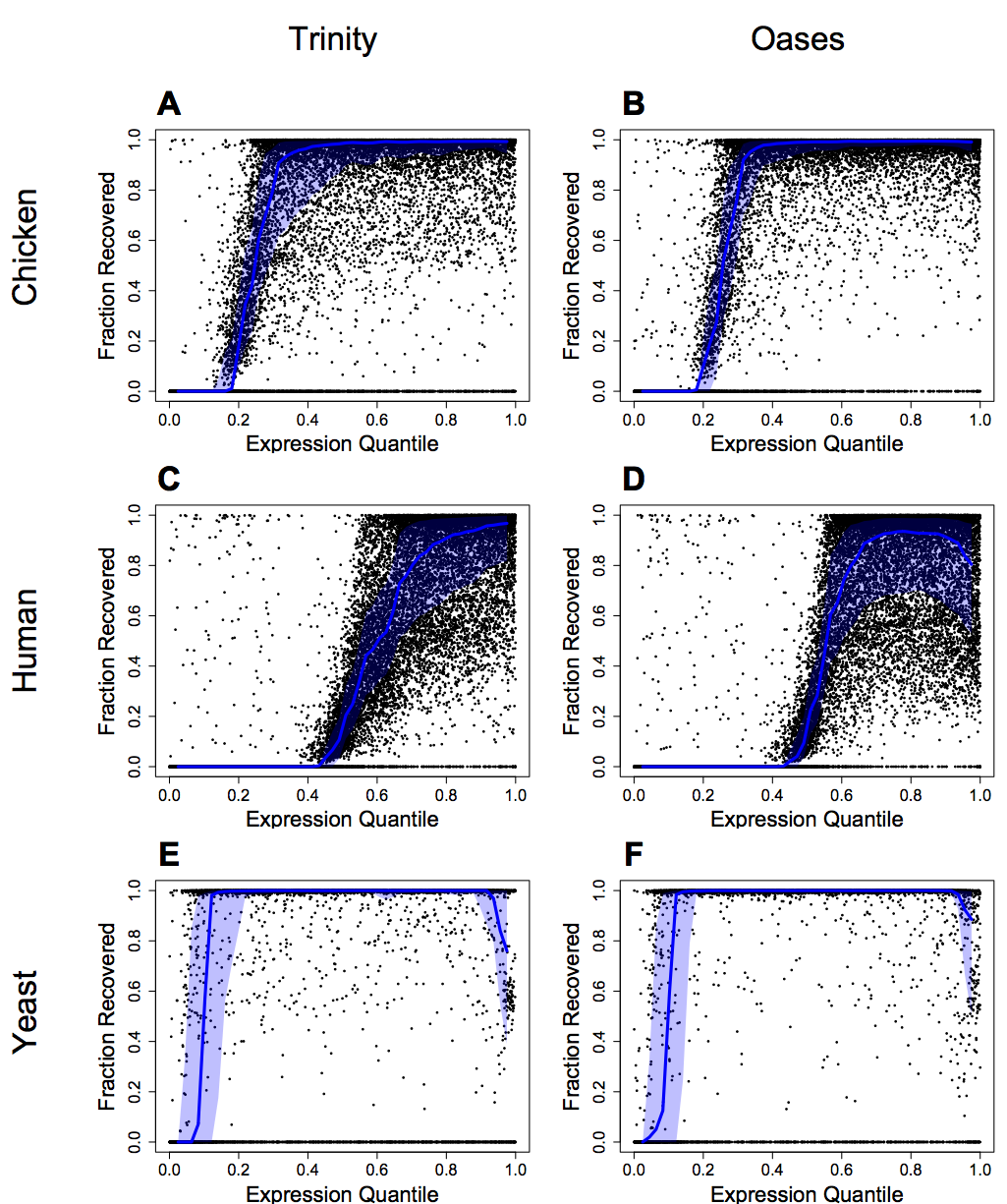
**

**Supplementary Figure 2: Metrics for assembly redundancy/fragmentation**

**A)** Histograms of the number of contigs assembled per gene (inset: y-axis on a logarithmic scale). Oases produced many more contigs per gene than Trinity. Even for the yeast dataset, which has minimal alternative splicing, Oases assembled many contigs per gene.

**B)** For the genes that had multiple contigs (and therefore required clustering) it was useful to assess the extent to which the contigs were redundant (for example the sequence of one contig was entirely contained within another). In these cases, it should be easy to cluster the contigs (see also Supplementary Figure 4). The converse situation can also occur, for example when there are gaps in the read coverage across a gene, and multiple non-overlapping contigs are assembled. In these cases the clustering algorithms will most likely fail to group the different fragments together. It is also possible that some contigs are partially overlapping, or that a gene with multiple assembled contigs will have some which are redundant, and some which are discontiguous. The frequency of these scenarios dictates how well contigs will be correctly clustered together (we refer to this as clustering recall, see the paper for a formal definition).

We have attempted to assess the frequency of these scenarios using a variable based on the amount of overlap between contig sequences. We call this the *average pairwise overlap*, defined for each gene as:

$$\frac{1}{number of pairs}\sum_{\mathrm{pairs}} \frac{Length of sequence overlap}{Length of shorter contig}$$

Where *pairs* refers to all possible pairs of assembled contigs matched to the gene. Average pairwise overlap is conceptually similar to the distances used by CD-HIT-EST and Corset.

We plot this quantity as a histogram (inset: y-axis on a logarithmic scale). This quantity tends to be either one (fully overlapping) or zero (no sequence in common). The chicken dataset has the potential for better clustering recall than the human dataset, because it has a higher ratio of fully overlapping to non-overlapping contigs. The Oases assembly for yeast also appears to produce many fully redundant contigs.

Note that the spikes in the logarithmic scale plots are cause by dividing discrete values. For example, the average pairwise overlap for a gene with three contigs, where two overlap completely and the third shares no sequence with the others would be 1/3.

**Supplementary Figure 3: Chimeric contigs in the assembly.**

Poor clustering precision is a consequence of “over-clustering” whereby contigs from different genes are grouped together. This happens when such contigs share sequence, such as paralogs, a common domain, overlapping UTRs or repeats. In some cases, overlapping sequence can also result in a chimeric contig being erroneously assembled.

We classified each contig as either “regular” (grey) – the contig sequence only matched one truth gene, “shared sequence” (green) – some sequence within the contig matched more than one gene (as would be expected for paralogs, common domains, repeats etc), “chimeric” (red) – the contigs contained sequence from multiple genes, and “multi-type” (blue) - where the contig belongs to both “chimeric” and “shared sequence”. We classified by aligning contigs against the annotated transcriptome using BLAT (requiring 200 bases with more than 98% identity). “shared sequence” contigs were differentiated from “chimeric”, as those where the annotated gene sequences overlapped by 100 or more bases. In A) we show the proportion of each contig type in the assembly. In B) we cluster together contig from the same gene, based on the truth mapping described in the previous plot. We then classify each gene according to its constituent contigs as: “regular” if all its contigs are “regular”, “similar sequence” if any of its contigs are “similar sequence”, “chimeric” if any of its contigs are “chimeric”, and “multi-type” if it has contigs from both “chimeric” and “similar sequence” classifications. Figure B) shows the proportion of genes of each type out of all genes present in the assembly.

We found that 20-80% of genes contained at least one contig that shared sequence with multiple genes. The proportion of “shared sequence” was similar for each dataset regardless of the assembly used, as would be expected for genome specific sequence overlaps such as paralogs, overlapping UTRs etc. In contrast, chimeric contigs were affected by both the assembler and dataset, consistent with chimeras being artifact of the assembly process. It is noticeable that *de novo* assemblies consist of a large number of contigs that are false chimeras. This is particularly true for gene dense genomes such as yeast.

**A) Contig type as a fraction of contigs.**

**B) Gene type as a fraction of genes**

**Section 2: Impact of *de novo* assembly quality on clustering**

In Supplementary Figure 4-6 below, we look at clustering recall and precision as a function of various aspects of the de novo assembly and read data. In Figure 2 of the paper, we define recall as true positives / (true positives + false negatives) and precision as true positives / (true positives + false positives). Where true positives are the number of pairs of contigs that are correctly clustered together, false positives are the number of pairs of contigs that are incorrectly clustered together etc.

For the plots below, we instead calculate the precision and recall for each gene separately, by defining true positives as the number of contigs from gene *g* which are correctly clustered, the number of false negatives as the number of pairs of contigs from gene *g* which are split into separate clusters, and false positives as the number of pairs of contigs incorrectly clustered together, where one contig in the pair is from gene *g*.

The recall/precisions values in the plots that follow show the mean recall/precision value for genes within that bin. Genes with a single contig that are clustered on their own, are not included in the recall/precision calculation.

**Supplementary Figure 4: How the “average pairwise overlap” affects clustering**

Clustering recall is affected by the degree of sequence overlap between contigs in a gene. Clustering together contig from the same gene that share no sequence is impossible (*average pairwise overlap* of zero). Contigs will remain in separate clusters giving a high false negative rate. When contigs share close to all their sequence the clustering become easier and the false negative rate will be low.

We examined how each clustering algorithm behaved as a function of *average pairwise overlap* (defined in Supplementary Figure 2), by binning the data in increments over this variable. The points show the mean recall value for each bin. Our method to calculate recall is described above. The vertical dashed line is the mean *average pairwise overlap* for the assembly.

The assemblers’ clustering consistently performed best in terms of Recall. CD-HIT-EST failed to cluster correctly even when the contigs were fully redundant. The trend in recall performance appears to be similar for all six datasets.

**Supplementary Figure 5: How clustering precision is affected by chimeric contigs and genes that share sequence.**

Shared sequence caused poor precision (or contigs from different genes to be clustered together). We examined how each clustering algorithm performed for each of our gene classes (defined in Supplementary Figure 3).

The Y-axis below shows the average clustering precision for each gene (described is more detail at the start of this section). Corset and CD-HIT-EST give prefect precision on genes without any shared sequence (“Regular”). For genes that shared sequence, CD-HIT-EST performs best, followed by Corset. The assemblers’ and in particular Oases give the poorest precision.

Note, in all clustering results shown in the paper and supplementary material we have ignored the chimeric contigs because they cannot be unambiguously associated to a single “truth” gene. Genes classified as “chimeric” have at least one matched “chimeric” contig, but may also have matched “regular” contigs. Hence, the precision results shown below for “chimeric” genes are calculated using only their “regular” contigs. If chimeric contigs were included the precision would likely be much lower, but we still see a loss of precision for these “regular” contigs that share sequence with “chimeric” contigs.

**Supplementary Figure 6: Clustering precision and recall as a function of expression quantile.**

The FPKM values from the genome-bases Cuffdiff analysis were used to order genes into expression quantiles. We used these expression quantiles to demonstrate the robustness of Corset clustering over a range of read coverages. In the figures below for A) Recall and B) Precision the range shown has been limited to the range where contigs were reconstructed (e.g. from Supplementary Figure 1).

**A) Recall**

Corset (pink) performs similarly to the assemblers’ clustering (grey) as a function of expression quantile. All methods show a drop in recall for lower expression quantile. This is presumably, because assembled genes have gaps in their sequence in these cases. There also appears to be some drop in the recall at the higher quantile end, perhaps because there are more contigs per gene in these cases, making it more difficult to cluster.

**B) Precision**

Corset (pink) performs similarly to CD-HIT-ESTs clustering (blue) as a function of expression quantile.

**Section 3: Supplementary data on Corset’s clustering**

**Supplementary Table 1: Removing contigs with low coverage**

By default, Corset will remove any contig with fewer than 10 reads aligning to it. This criterion has an impact on the final number of contigs and clusters reported by Corset. For the human RNA-Seq dataset assembled with Trinity, we examined the effect of altering the minimum reads threshold.

The table below shows the minimum reads threshold we applied, the number of contigs that pass this threshold, the number of clusters reported by Corset, and the number of known genes that are represented by the reduced set of contigs (according to BLAT alignment of known genes to assembled contigs).

The number of genes represented in the final set of contigs decreases by over 700, however it should be noted that significant differential expression can not be detected from amongst these genes. By applying the default threshold, the number of clusters is approximately halved and the number of contigs is reduced by almost 40 thousand.

| **Read**  **Threshold:** | **0** | **1** | **3** | **5** | **8** | **10** |
| --- | --- | --- | --- | --- | --- | --- |
| Contigs | 107,389 | 102,650 | 92,642 | 83,206 | 73,466 | 69,107 |
| Corset  Clusters | 79,979 | 75,393 | 65,761 | 56,773 | 47,665 | 43,663 |
| Known Genes after Corset clustering | 12,891 | 12,826 | 12,677 | 12,480 | 12,281 | 12,160 |

**Supplementary Figure 7. The effect of different p-value thresholds when testing pairs of contigs for proportional expression**.

The cumulative number of true positive differentially expressed clusters against the number of top ranked clusters is shown. A core component of the Corset clustering algorithm is a likelihood ratio test to separate paralogs and differently expressed isoforms, based on contig pairs having unproportional expression. The black curve shows results including the test for proportionality while the blue line shows results without the test. Differential expression results for a range of p-value thresholds are shown (orange shaded region), demonstrating that the test is robust to the choice of threshold. See the description of Figure 3 in the main paper for more details.

**
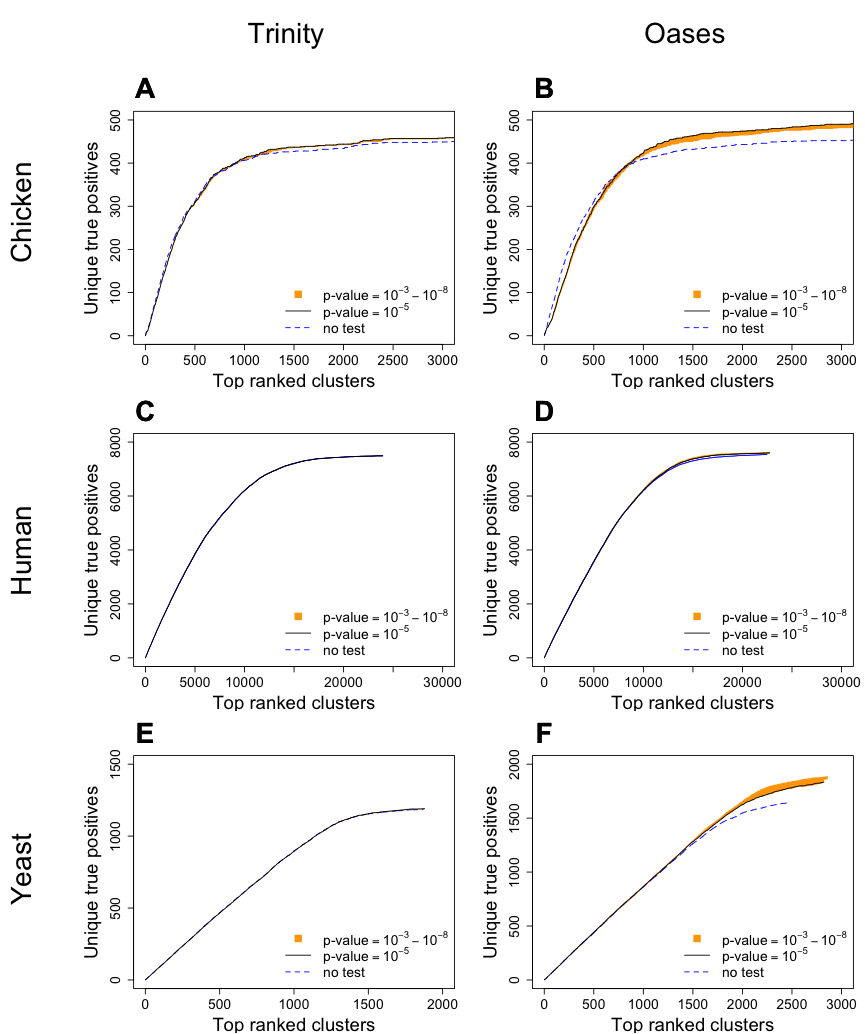
**

**Supplementary Figure 8. The precision and recall for various clustering options.**

For the hierarchical clustering in Corset**,** different distance thresholds between 0.1 and 0.9 were used. The results were robust to the choice of distance. See the description of Figure 2 in the paper for more detail. We show the results for six different assemblies: a) chicken data assembled with Trinity, b) chicken data assembled with Oases, c) human data assembled with Trinity, d) human data assembled with Oases, e) yeast data assembled with Trinity and f) yeast data assembled with Oases. The X indicates perfect clustering.

**Supplementary Figure 9. Robustness of DGE results with respect to the clustering distance threshold.**

The cumulative number of true positive differentially expressed clusters against the number of top clusters is shown. We varied the clustering distance threshold between 0.1 and 0.9 (orange shaded region), and found the differential gene expression results to be robust with respect to the distance threshold. The default clustering which uses a threshold distance of 0.3 (black curve) is shown for comparison. See Figure 3 in the paper for more detail.


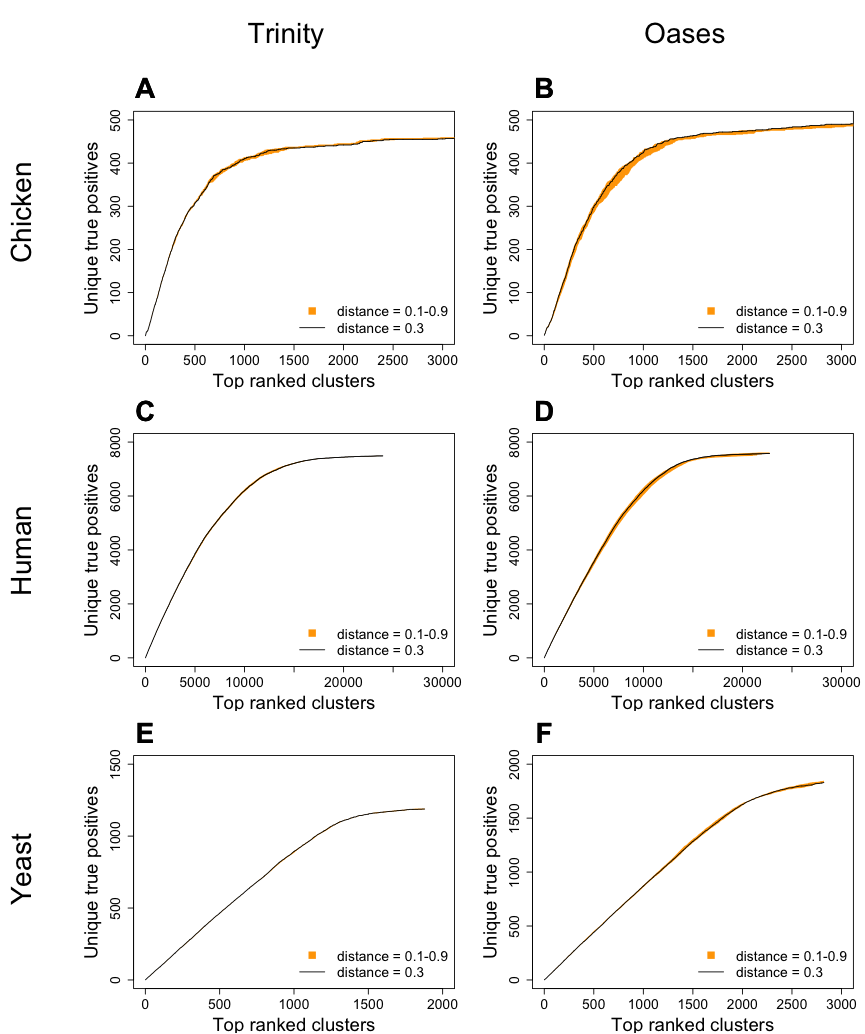


**Supplementary Figure 10. DGE results with truth defined by edgeR.**

The cumulative number of true positive differentially expressed clusters against the number of top clusters is shown. This is similar to Figure 4 in the manuscript, but edgeR is used to define true differential expression rather than cuffdiff2. Although, the number of true positives is markedly different between cuffdiff2 and edgeR, the performance of Corset, Trinity, Oases, CD-HIT-EST and no clustering, are similar relative to one another. Ideal clustering referers to “truth” clustering, in which the clusters are defined using truth information about mappings of genes to contigs.

**Supplementary Section 4: results for abundance estimation**

We compared four pipelines for calculating cluster-level counts against Corset: RSEM, mapping to the longest contig in each cluster, and single-mapping then summation (see Methods in the paper). We used Corset for defining clusters, however the default behavior of filtering low expressed transcripts was switched off in all cases. This was done because RSEM will not run with a cluster list where some transcripts have been filtered out. However, we later removed clusters if no counting method reported 10 or more total counts (summed across samples). All counting methods produced similar results (Supplementary Tables 2 and 3), but we found a hint that RSEM underestimates counts for a small fraction of clusters (Supplementary Table 4 and 5, and Supplementary Figure 11 and 12).

**Supplementary Table 2. The Pearson correlation between log2 Corset counts and log2 counts from other methods.** For each counting method (rows) and each assembly (columns) we calculated the average cluster-level counts for each experimental group. We then compared these values against those obtained from Corset. The Pearson correlation is high in all cases, however RSEM is consistently lower than other methods. This is driven by RSEM estimating lower counts that Corset for a small number of groups (Supplementary Figure 11).

|  | Chicken | | Human | | Yeast | |
| --- | --- | --- | --- | --- | --- | --- |
|  | Trinity | Oases | Trinity | Oases | Trinity | Oases |
| RSEM | 0.990 | 0.953 | 0.997 | 0.985 | 0.969 | 0.767 |
| Single Map & Sum | 0.999 | 0.993 | 1.000 | 0.999 | 1.000 | 0.979 |
| Longest | 0.998 | 0.996 | 1.000 | 0.997 | 1.000 | 0.994 |

**Supplementary Table 3. The Similarity between Corset counts and counts from other methods.** As in Supplementary Table 2, for each counting method (rows) and each assembly (columns) we calculated the average cluster-level counts for each experimental group. Below, we show the percentage of values which were identical to Corset’s estimates or within 10% of Corset’s estimates (in brackets). The consistency between methods is generally high, in particular for Trinity assemblies.

|  | Chicken | | Human | | Yeast | |
| --- | --- | --- | --- | --- | --- | --- |
|  | Trinity | Oases | Trinity | Oases | Trinity | Oases |
| RSEM | 93 (96) | 71 (80) | 95 (98) | 81 (90) | 94 (96) | 56 (67) |
| Single Map & Sum | 92 (96) | 71 (82) | 95 (98) | 80 (92) | 94 (99) | 57 (72) |
| Longest | 84 (91) | 60 (77) | 82 (96) | 57 (84) | 91 (98) | 41 (86) |

**Supplementary Figure 11. Discrepancy in cluster-level counts between RSEM and Corset on the human dataset assembled with Trinity.** For both Corset and RSEM we calculated the average cluster-level counts for each experimental group. While 95% of values were in complete agreement, for a small number of values, large discrepancies were seen in which RSEM reported fewer or no counts compared to Corset. A) Shows the ratio of log2 counts for RSEM to Corset as a function of the counts averaged between RSEM and Corset. Values in agreement were excluded from the plot. B) For the 5% of values where there was a discrepancy in counts, we calculated the coefficient of variation between biological replicates. Corset was found to be more consistent between replicates. Here we have shown results for the human dataset assembled with Trinity, however underestimation of counts by RSEM was seen for all assembled transcriptomes.

**Supplementary Table 4. The variation between biological replicates for RSEM and Corset**. For each group, where the average cluster-level counts from RSEM disagreed with Corset, we calculated the coefficient of variation for biological replicates within the group. Shown below is the average coefficient of variation across groups. Corset’s count estimates show lower variation within experimental groups, indicating that they may be more accurate.

|  | Chicken | | Human | | Yeast | |
| --- | --- | --- | --- | --- | --- | --- |
|  | Trinity | Oases | Trinity | Oases | Trinity | Oases |
| Corset | 0.27 | 0.26 | 0.29 | 0.27 | 0.23 | 0.24 |
| RSEM | 0.33 | 0.35 | 0.36 | 0.32 | 0.25 | 0.30 |

To investigate the discrepancy between Corset and RSEM further we performed a truth based analysis. For this analysis, we did the following:

1. We switched to using “Ideal” clustering to reduce the chance of bias from the choice of clustering. For “Ideal” clustering, contigs were assigned to groups corresponding to their aligned gene.
2. We defined “true” counts by running RSEM on transcript sequences from the reference annotation. Reads were mapped as defined in the methods, and RSEM was run with default settings.
3. We then compared the following three pipelines:
   1. Multi-mapping reads to the reference annotation sequence followed by running RSEM (denoted as *Truth*)
   2. Multi-mapping to the assembled contigs, followed by running RSEM (denotes as *RSEM*)
   3. Mapping to the assembled contigs (but only allowing a single hit), aggregating the counts to cluster-level. This is the single-map & sum method described in the methods part of the paper. It is conceptually similar to what Corset does and gives similar values (denoted *Corset-style)*. We used this because Corset counts can only be obtained for corset clustering.

Supplementary Figure 12 shows the results for the chicken-Oases dataset. A) there is a bias in RSEM counts when run on the assembly and B) this results in missed true positive differentially expressed genes. This analysis was repeated for all six dataset, with similar observations. The Pearson correlations for the log_2_ count data are given in Supplementary Table 5.

**Supplementary Figure 12: RSEM performance for “Ideal” clustering**

For the chicken dataset assembled with Oases, we show A) the correlation between gene-level counts for Oases + RSEM (*RSEM*), compared to Annotation + RSEM (*Truth*) and Oases + single-map & sum (*Corset-style*). A half count offset is applied to all count values so their log is defined. *Corset-style* shows a stronger Pearson correlation against *Truth*, than *RSEM* against *Truth*, but *RSEM* and *Corset-style* are the most highly correlated (0.96). This result is driven by RSEM severely underestimating the counts in a number of cases. These outliers give rise to the false negatives observed in B) the cumulative number of unique true positives as a function of top ranked clusters. Here, true positives are defined by the Annotation + RSEM (*Truth*) counts analysed in edgeR.

**A) Concordance of count data
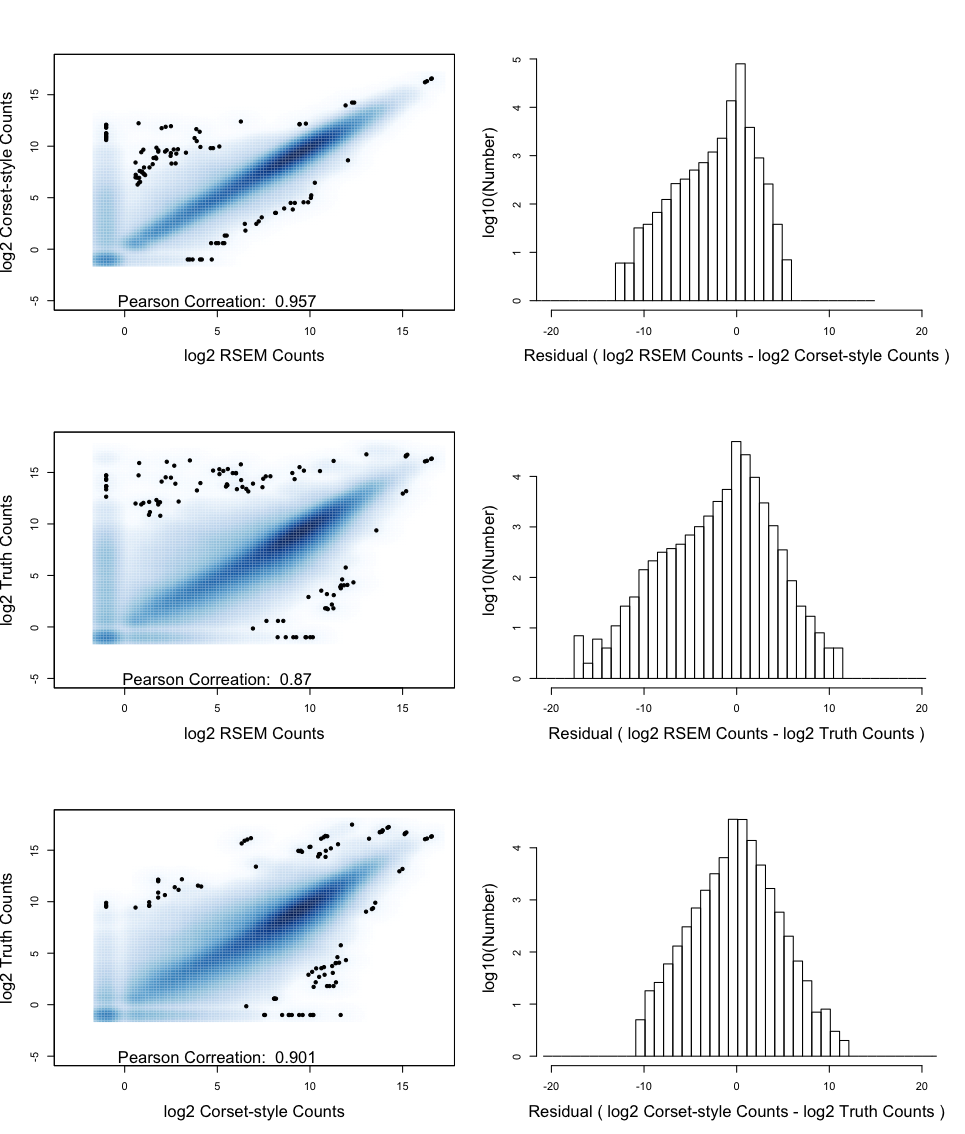
**

1. **Cumulative number of unique true positives**

**Supplementary Table 5: Pearson correlations of log2 counts for all dataset**

For all dataset, we compared gene-level counts from Assembly + RSEM (*RSEM*) with Annotation + RSEM (*Truth*) and Assembly + single-map & sum (*Corset-style*). A half count offset was applied to all count values so their log was defined. *Corset-style* showed a stronger Pearson correlation against *Truth*, than *RSEM* against *Truth*, in all dataset apart from human-Trinity, where the correlation is the same. In all cases apart from the yeast assembled with Trinity, *RSEM* and *Corset-style* counts were extremely highly correlated (above 0.96).

|  | Chicken | | Human | | Yeast | |
| --- | --- | --- | --- | --- | --- | --- |
|  | Trinity | Oases | Trinity | Oases | Trinity | Oases |
| Corset-style versus Truth | 0.91 | 0.90 | 0.92 | 0.92 | 0.69 | 0.75 |
| RSEM versus Truth | 0.88 | 0.87 | 0.92 | 0.90 | 0.53 | 0.73 |
| RSEM versus Corset-style | 0.98 | 0.96 | 0.99 | 0.98 | 0.77 | 0.96 |
